# Enzymatic Polymerization on DNA Modified Gold Nanowire for Label-Free Detection of Pathogen DNA

**DOI:** 10.3390/ijms160613653

**Published:** 2015-06-15

**Authors:** Jaepil Jeong, Hyejin Kim, Jong Bum Lee

**Affiliations:** Department of Chemical Engineering, University of Seoul, Seoul 130-743, Korea; E-Mails: jpjeong@uos.ac.kr (J.J.); ajin2010@uos.ac.kr (H.K.)

**Keywords:** biosensor, label-free, pathogen DNA detection, rolling circle amplification (RCA), gold nanowire

## Abstract

This paper presents a label-free biosensor for the detection of single-stranded pathogen DNA through the target-enhanced gelation between gold nanowires (AuNW) and the primer DNAs branched on AuNW. The target DNA enables circularization of the linear DNA template, and the primer DNA is elongated continuously via rolling circle amplification. As a result, in the presence of the target DNA, a macroscopic hydrogel was fabricated by the entanglement of the elongated DNA with AuNWs as a scaffold fiber for effective gelation. In contrast, very small separate particles were generated in the absence of the target DNA. This label-free biosensor might be a promising tool for the detection of pathogen DNAs without any devices for further analysis. Moreover, the biosensor based on the weaving of AuNW and DNAs suggests a novel direction for the applications of AuNWs in biological engineering.

## 1. Introduction

Deoxyribonucleic acid (DNA) has significant potential as a multifunctional material, and its programmability through base-paring of DNA [1,2] has enabled the use of DNA as a building block. By taking advantage of DNA as a versatile material, a wide range of applications have been introduced, such as drug delivery [3,4], pathologic diagnosis [5,6], microscale patterning [7,8], arraying nanomaterials [9,10], and construction of structures in various shapes [11,12,13]. Furthermore, the conjugation of DNA with various materials, such as gold [14,15], silver [16,17], and magnetic core [18], have endowed additional functions to the DNA nanostructures. Among them, gold has been adopted widely for its optical and photothermal properties induced by its localized surface plasmon resonance in a variety of shapes, such as nanoparticles [19,20], nanorods [21], nanoplate films [22], and nanoclusters [23]. To date, however, there are few reports on the use of gold nanowires (AuNWs) in biological engineering. In addition, AuNWs in the majority of the studies focused on gold nanoparticles assembled in a line rather than gold nanowires per se [24,25,26].

This paper proposes a label-free biosensor for single-stranded DNA detection induced by the interlacing of AuNW and primer DNA branched on the AuNW. The rolling circle amplification (RCA) technique was used on the branched primer DNA. RCA is an isothermal and enzymatic process, which enables the continuous amplification of DNA sequences [27,28,29]. In the present study, macroscopic hydrogel was produced via the entanglement of elongated primer DNA and AuNWs in the presence of the target pathogen DNA. To the best of the authors’ knowledge, this is the first approach of the use of AuNWs as a scaffold fiber for effective gelation. Using this biosensor, the existence of pathogen DNA was distinguishable by naked eye, which may offer a novel way for point-of-care diagnosis.

## 2. Results and Discussion

### 2.1. Design of AuNW: DNA Based Biosensor

**Figure 1:**
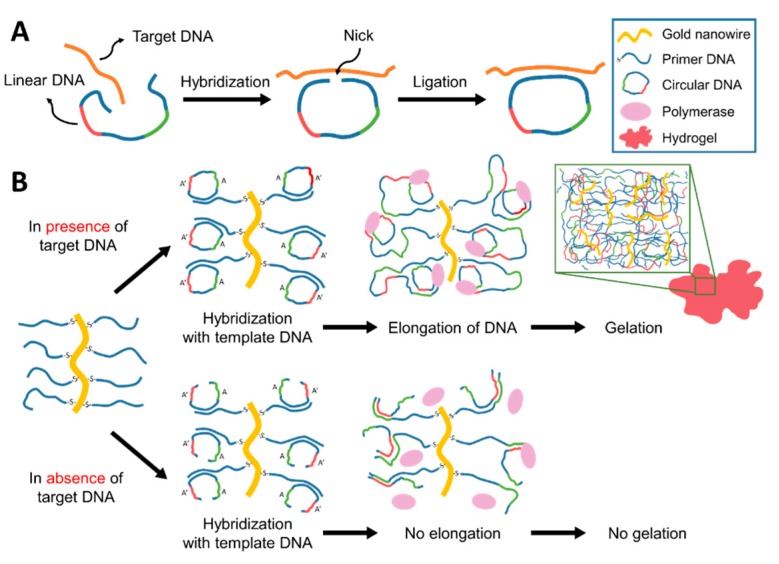
Schematic illustration of the label-free detection of single-stranded DNA. (**A**) Scheme of circular DNA preparation in the presence of target DNA; and (**B**) scheme of hydrogel synthesis with the prepared circular DNA. S indicates thiol group. Sequence of A and Aʹ are complementary.

To synthesize the AuNW: DNA based biosensor, the target DNA was first designed from influenza A virus DNA. Subsequently, 13 bases from the 5′ end and 14 bases from the 3′ end of the linear DNA were designed to be complementary to the 27 bases of the target DNA, leaving a few bases at both ends. By rational design, the linear DNA can be circularized in the presence of the target DNA by temperature annealing, as shown in Figure 1A. The discontinuous region between the 5′ and 3′ ends of the linear DNA was connected enzymatically by ligation, and closed circular DNA was formed. On the other hand, in the absence of the target DNA, the complete circularization of linear DNA is impossible, even after ligation.

The 5′ end of primer DNA was modified chemically with an alkane thiol group to immobilize the primer DNA on AuNW, as shown in Figure 1B. After removing the excess primer DNA, the primer DNA immobilized on AuNW was allowed to hybridize with the linear or circular DNA for the initiation of RCA. Moreover, 12 bases (denoted as A) and the other 12 bases (denoted as A′) of the linear DNA were designed complementary to each other, which enabled efficient cross-linking.

**Figure 2:**
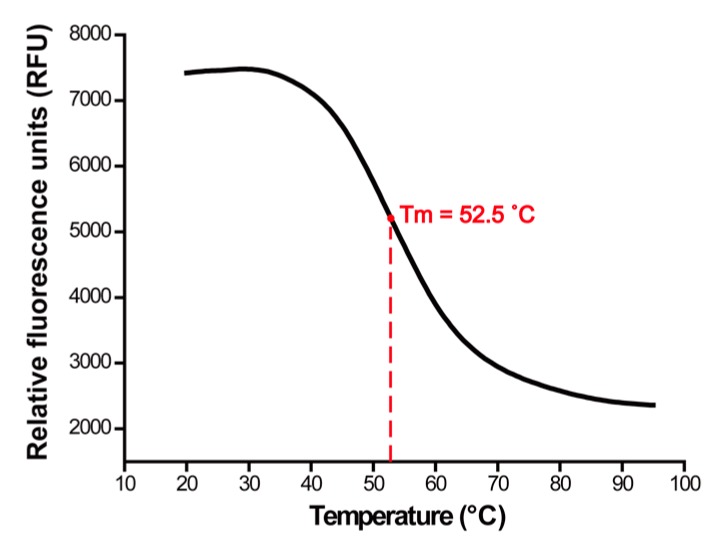
Melt curve of the primer DNA modified on gold nanowires. The temperature was increased gradually by 0.5 °C from 20 to 95 °C.

Because the surface of AuNW is covered with cationic cetyltrimethylammonium bromide (CTAB) molecules, it is essential to determine if the primer DNAs are functionalized on AuNWs by sulfur-gold bonding or electrostatic interactions due to the negatively charged nature of DNA. To examine the binding characteristic between the primer DNA and AuNW, the melting temperature between the primer DNAs on the AuNW and the linear DNAs were investigated using the DNA-intercalating fluorescence dye. A sudden decrease in the fluorescence intensity on the melt curve shown in Figure 2 suggests that the primer DNAs is immobilized on the AuNW through a sulfur-gold interaction. As the charge-induced binding between DNA and AuNW is irregular, programmed assembly between the primer DNA and linear DNA would be inhibited, and randomly denatured DNA would result in a gradual decrease in the fluorescence intensity on the melt curve rather than a sudden decline [30].

To demonstrate the target-enhanced gelation via rolling circle amplification, the previously circularized DNA template was allowed to hybridize with the primer DNA modified on AuNW. As the 3′ end of the target DNA is designed not to hybridize with linear DNA, the 3′ end of the primer DNA is the only initiation site for phi29 DNA polymerase. As shown in Figure 1B, the DNA strands were elongated continuously via RCA from the previously synthesized circular DNA in the presence of the target DNA. The AuNW is believed to work as a scaffold and amplified DNA strands on AuNW function as the side chains that facilitate expeditious gelation. In absence of the target DNA, however, RCA was not performed with the unligated linear DNA. Therefore, the primer DNA could not be elongated. As a result, macroscopic hydrogel is synthesized only in the presence of the target DNA. Also, same results were obtained when reacson was performed with library of non-complementary DNA, in the presence (see Figure S1) or absence of target DNA (see Figure S2), which implies potential for selective detection.

### 2.2. Characterization of AuNW: DNA Based Biosensor

**Figure 3:**
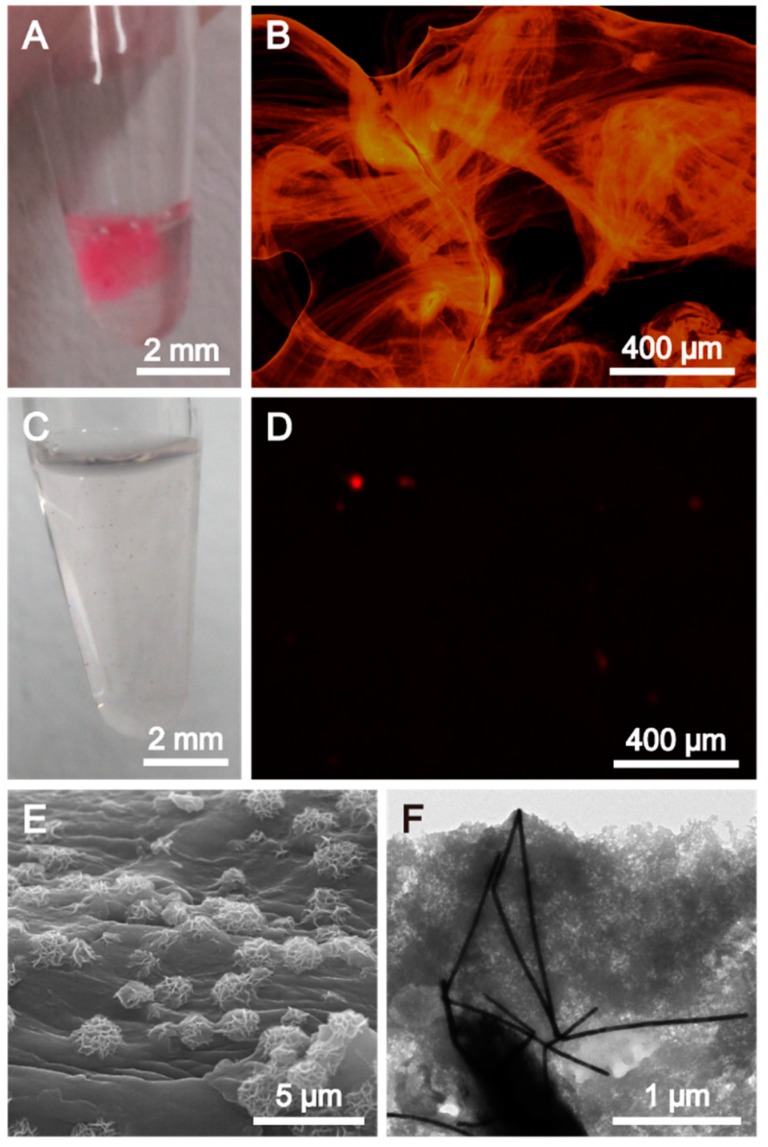
(**A–D**) Digital camera images and fluorescence microscopy images of the products of rolling circle amplification in the presence (**A**,**B**) and absence (**C**,**D**) of the target DNA; (**E**) SEM and (**F**) TEM images of the AuNW-DNA hydrogel after rolling circle amplification with the target DNA.

After the RCA process was conducted on the AuNW: DNA based biosensor, the products were analyzed to confirm the potential utilization of the AuNW: DNA based biosensor for pathogen DNA detection. The digital camera image of the RCA product shown in Figure 3A showed that the hydrogel formed in the presence of the target DNA is apparently visible to the naked eye. In contrast, no macroscopic structures were observed in the absence of the target DNA (Figure 3C). After staining DNA hydrogel with DNA specific dye (GelRed), strong fluorescence from hydrogel also confirmed that the resulting product was made of DNA (Figure 3B). Moreover, the fluorescence microscopic image of the stained hydrogel showed that many thin DNA film-like structures were closely packed and interlaced with each other in the presence of the target DNA (Figure 3B). However, only small features were observed in the absence of the target DNA (Figure 3D). The microscopic structures of the RCA products in the presence of target DNA were investigated further by scanning electron microscopy (SEM) and transmission electron microscopy (TEM). The surficial structure of the hydrogel was decorated with porous ball-like particles (Figure 3E). The TEM image demonstrated in Figure 3F showed that DNAs were entangled with the gold nanowires as a result of the crosslinking of elongated DNA.

## 3. Experimental Section

### 3.1. Materials

All DNA oligonucleotides (Primer DNA: 5′ SH-TTT TTT TTT TTT TTT TTT TTA CGA CGT GTG ACC ATG CA-3′; Linear DNA: 5′-ACT TGC GGC AAT ACA AGT CGT CTC GTC GCA CTC TTT TTG CAT GGT CAC ACG TCG TTC TAT TGT GCG ACG AGA CCG TTT CAA GAT CCC AAT GAT-3′; Target DNA: 5′-TGT TAT TGC CGC AAG TAT CAT TGG GAT CTT GCA CTT-3′) were purchased from IDT. The target DNA used in this study was formulated from the influenza A virus sequences shown in Genbank database (http://www.ncbi.nlm.nih.gov/nuccore/401716582, accessed 20 March 2014). Dithiothreitol (DTT) and gold nanowires (AuNW) were purchased from Sigma-Aldrich (Saint Louis, MO, USA). NAP-5 column (Sephadex G-25 DNA grade) was purchased from G.E. Healthcare (Amersham, UK). T4 DNA Ligase and 10× ligase buffer were purchased from Promega (Madison, WI, USA). Phi29 DNA polymerase, 10× phi29 buffer and deoxyribonucleotide triphosphate mixture (dNTPs) were purchased from Epicentre (Madison, WI, USA). Evagreen was purchased from Biotium (Carlsbad, CA, USA).

### 3.2. Preparation of Thiolated Oligonucleotide-Modified Gold Nanowires

The thiolated primer DNAs were deprotected by addition of 0.2 M DTT, 0.18 M phosphate buffer (pH 8.0) and incubated at room temperature for 2 h. The primer DNAs were then washed over a NAP-5 desalting column to remove DTT. Purified primer DNAs (final concentration of 1.875 μM) were then mixed with AuNW, phosphate (final concentration of 10 mM) and NaCl (1 M). After 20 h incubation at room temperature, the solution was centrifuged at 13,000 rpm for 20 min, and the supernatant was discarded for the removal of unconjugated primer DNAs. This washing step was repeated 4 times. Finally, the AuNW-Primer DNA conjugates (AuNW:Primer) were redispersed in nuclease-free water.

### 3.3. Procedure for the Label-Free Detection of Target DNA

To produce the circularized DNA, the linear DNA and target DNA were mixed in nuclease-free water at a final concentration of 7.5 μM each. Temperature annealing was performed for the efficient hybridization between the linear DNA and target DNA. The mixture was heated to 95 °C for 2 min, then cooled gradually to 25 °C over a 60 min period. After 20 min incubation at 25 °C, the annealed DNA was mixed with T4 DNA ligase (3 U·μL^-1^) and ligase buffer (300 mM Tris-HCl (pH 7.8), 100 mM MgCl_2_, 100 mM DTT and 10 mM ATP) and incubated overnight at room temperature to close the nick.

Rolling circle amplification (RCA) was then performed as follows. First, 20 μL of the closed circular DNA (final concentration of 3 μM) and AuNW:Primer (30 μL) were combined and incubated for 2 h at room temperature for hybridization between the circular DNA and the primer DNA on AuNW. After incubation, unhybridized circular DNA was removed by discarding the supernatant after centrifugation at 13,000 rpm for 20 min. After washing twice, the resulting product was mixed with phi29 DNA polymerase (10 U·μL^−1^), phi29 reaction buffer (80 mM Tris-HCl (pH 7.5), 100 mM KCl, 20 mM MgCl_2_, 10 mM (NH_4_)_2_SO_4_ and 8 mM DTT) and dNTPs (2 mM). Finally, the solution was incubated at 30 °C for 20 h.

### 3.4. Analysis of Characteristics of the Target-Enhanced Gelation

The melt curve was acquired using a CFX96 Touch Real-Time PCR Detection System (Bio-Rad, Foster, CA, USA). The linear DNA (final concentration of 2.5 μM) and AuNW:Primer (10 μL) was combined and incubated for 2 h at room temperature for hybridization. After washing twice, as mentioned above, NaCl (final concentration of 50 mM) and Evagreen were added. After 20 min incubation at 20 °C, the solution was heated gradually to 95 °C at a rate of 0.5 °C/min.

To obtain the fluorescence images of the hydrogel, the hydrogel was stained with GelRed and incubated overnight. After washing thoroughly, the fluorescence images were acquired by Eclipse Ti-U (Nikon, Tokyo, Japan) inverted fluorescent microscopy. A scanning electron microscopy (SEM) image was acquired using FE-SEM (S-4200, Hitachi, Tokyo, Japan) and the sample was coated with Pt. Cryo TEM (Cryo Tecnai F20 G2, FEI, Hillsboro, OR, USA) was used to obtain a TEM image, and the grid was treated with oxygen plasma.

## 4. Conclusions

A biosensor based on interlacing of DNA modified AuNW was developed for the detection of single-stranded pathogen DNA. Primer DNA coated AuNW formed a macroscopic hydrogel by simple hybridization and RCA. After the RCA process performed on the biosensor, enhanced gelation was observed in the presence of pathogen DNA. Although more studies on detecting various targets like protein or cell using target-binding molecules like aptamer are needed, it is believed that the proposed biosensor can enable label-free and easily recognizable detection without complicated processes or equipment.

## Supplementary Materials

Supplementary materials can be found at: http://www.mdpi.com/1422-0067/16/06/13653/s1.
