# Supplementary material for: Enzymatic Polymerization on DNA Modified Gold Nanowire for Label-Free Detection of Pathogen DNA
Source: Int J Mol Sci. 2015 Jun 15;16(6):13653–60. doi: 10.3390/ijms160613653 (PMC4490515; doi:10.3390/ijms160613653)
Supplement: Supplementary file 1 [file ijms-16-13653-s001.pdf]

Supplementary Information

1. Supplementary Experimental Section

1.1. Materials

All DNA oligonucleotides (H1N1 DNA: 5′ ATT CAA ATG GAA CCG TCA AGG-3′; HIV DNA: 5′-GAC TCA GAT TGG TTG CAC TTT-3′; Scramble DNA: TAA TAC GACTCA CTA TAG GGA-3′) were purchased from IDT.

1.2. Detecting Target DNA in the Presence of a DNA Library

For the selectivity study, circularized DNA was produced in the presence of a library of non-complementary DNA. The linear DNA, target DNA, H1N1 DNA, HIV DNA and Scramble DNA were mixed in nuclease-free water at a final concentration of 7.5 μM each and temperature annealing was followed. The mixture was heated to 95 °C for 2 min, then cooled gradually to 25 °C over a 60-min period. After 20 min incubation at 25 °C, the annealed DNA was mixed with T4 DNA ligase (3 $U \cdot \mu L^{-1}$) and ligase buffer (300 mM Tris-HCl (pH 7.8), 100 mM $MgCl_2$, 100 mM DTT, and 10 mM ATP) and incubated overnight at room temperature. RCA was performed in same way as written in the Experimental Section of this article.

1.3. Control Experiment in the Presence of a Library of Non-Complementary DNA

For the control experiment, linear DNA, H1N1 DNA, HIV DNA and Scramble DNA were mixed in nuclease-free water at a final concentration of 7.5 μM each and temperature annealing was followed. The mixture was heated to 95 °C for 2 min, then cooled gradually to 25 °C over a 60-min period. After 20 min incubation at 25 °C, the annealed DNA was mixed with T4 DNA ligase (3 $U \cdot \mu L^{-1}$) and ligase buffer (300 mM Tris-HCl (pH 7.8), 100 mM $MgCl_2$, 100 mM DTT, and 10 mM ATP) and incubated overnight at room temperature. RCA was performed in same way as written in the Experimental Section of this article.

2. Detecting Target DNA in the Presence of DNA Library

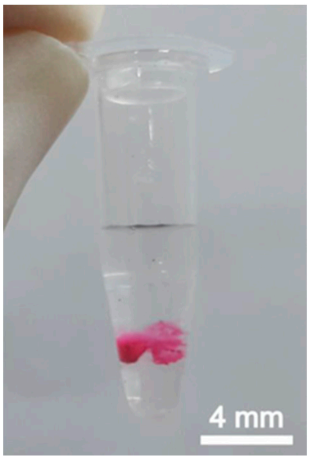

Figure S1. Digital camera image of the product of rolling circle amplification in the presence of target DNA and library of non-complementary DNA.

3. Control Experiment in the Presence of DNA Library

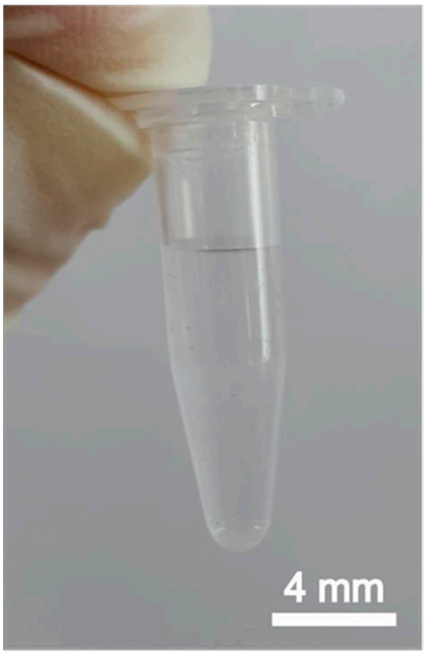

Figure S2. Digital camera image after 20 h of rolling circle amplification with library of non-complementary DNA in the absence of target DNA.
